# Comparative Carcinogenic Risk Assessment of Polycyclic Aromatic Hydrocarbons Accumulation in Normal and Cancerous Lung Tissues of Patients from Northern Thailand

**DOI:** 10.3390/toxics14080702

**Published:** 2026-08-09

**Authors:** Nuttipon Yabueng, Yanisa Samakarn, Kavee Pairojtanachai, Pavidarin Kraisitnitikul, Jaturawit Kabao, Pattraporn Tajarernmuang, Atirut Supphapipat, Sophon Siwachat, Somcharoen Saeteng, Somporn Chantara, Apichat Tantraworasin

**Affiliations:** 1 Environmental Science Research Center, Faculty of Science, Chiang Mai University, Chiang Mai 50200, Thailand; nuttipon.y@cmu.ac.th (N.Y.); jaturawit_kj@hotmail.com (J.K.); 2 Faculty of Medicine, Chiang Mai University, Chiang Mai 50200, Thailand; yanisa.s@cmu.ac.th (Y.S.); kavee.pairojtanachai@cmu.ac.th (K.P.); 3 Office of Research Administration, Chiang Mai University, Chiang Mai 50200, Thailand; ppavidarinaor@gmail.com; 4 Department of Internal Medicine, Faculty of Medicine, Chiang Mai University, Chiang Mai 50200, Thailand; pattraporn.t@cmu.ac.th; 5 Clinical Surgical Research Center, Chiang Mai University, Chiang Mai 50200, Thailand; atirut_s@cmu.ac.th (A.S.); sophon.s@cmu.ac.th (S.S.); somcharoen_s19@outlook.com (S.S.); 6 Department of Surgery, Faculty of Medicine, Chiang Mai University, Chiang Mai 50200, Thailand; 7 Chemistry Department, Faculty of Science, Chiang Mai University, Chiang Mai 50200, Thailand

**Keywords:** lung cancer, PAHs, smoking, environmental exposure, air pollution

## Abstract

Lung cancer mortality is a critical issue in Northern Thailand, where incidence rates significantly exceed the national average. Key risk factors include tobacco smoking and seasonal air pollution from biomass burning, which releases carcinogenic polycyclic aromatic hydrocarbons (PAHs). This study aims to analyze the accumulation of 16 priority PAHs in human lung tissues to explore links between environmental exposure, smoking habits, and cancer development. Tissue samples (normal, para-cancerous, and adenocarcinoma) were collected from 30 surgical participants (15 lung cancer patients, 15 non-cancer controls) at Chiang Mai University Hospital. Compounds were extracted via ultrasonication and quantified using gas chromatography-mass spectrometry. Carcinogenic potency was evaluated using Benzo[a]pyrene equivalents, with concentrations compared across tissue types and patient groups using generalized estimating equations. High-molecular-weight PAHs were the dominant species, accounting for over 50% of total accumulation in all tissues, aligning with regional biomass burning profiles. Total concentrations and carcinogenic potency were significantly higher in smokers, particularly within normal lung tissues. Furthermore, high-molecular-weight PAH concentrations positively correlated with tissue cholesterol levels (R = 0.66). Source analysis indicated exposure primarily from pyrogenic sources, alongside petroleum emissions. In conclusion, high-molecular-weight carcinogens significantly accumulate in human lungs in Northern Thailand, reflecting substantial environmental exposure, while tissue lipid content modulates toxin bioaccumulation. Smoking facilitates compound retention in normal tissues, underscoring an urgent need for targeted public health strategies addressing both environmental pollution and tobacco use.

## 1. Introduction

Cancer remains a leading cause of death worldwide, accounting for nearly 10 million deaths—or approximately one in six deaths—in 2020 [1]. Among all malignancies, lung cancer is one of the most frequently diagnosed cancers and remains the leading cause of cancer-related mortality globally among both men and women [2,3,4,5]. In Thailand, lung cancer ranks second in both incidence and mortality, surpassed only by liver cancer. Notably, Northern Thailand exhibits the highest annual mean age-standardized incidence rates (ASR) in the country, reaching 24.7 per 100,000 for men and 15.5 per 100,000 for women in 2025—rates that are 1.2 and 1.6 times higher than the respective national averages [6].

Air pollution and tobacco smoking are well-established environmental risk factors for lung carcinogenesis [7,8,9]. In Northern Thailand, severe ambient air pollution recurs annually during the dry season (typically February through April), primarily driven by widespread biomass burning from forest fires and agricultural practices, which accounts for approximately 50% of the ambient fine particulate matter (PM2.5) mass [10,11]. During these peak months, daily average PM2.5 concentrations frequently exceed the national ambient air quality standard of Thailand ($37.5\mu \;{g/m}^{3}$) by 1.3- to 2.0-fold (http://air4thai.pcd.go.th/webV3/#/Home (accessed on 24 May 2026)). Notably, atmospheric PM2.5 levels in this region demonstrate a strong positive correlation (r=0.60−0.90) with carcinogenic polycyclic aromatic hydrocarbons (PAHs) [11,12]. In parallel, tobacco smoke represents another major source of toxic inhalation, releasing complex mixtures of metals, nicotine, aromatic amines, nitrosamines, phenols, BTEX (Benzene-Toluene-Ethylbenzene-Xylene), and PAHs [13,14,15].

PAHs are pervasive environmental pollutants generated through the incomplete combustion of organic materials, including fossil fuels, wood, and biomass [16,17]. They are of paramount public health concern due to their environmental persistence, tissue bioaccumulation, and well-documented mutagenic, carcinogenic, and teratogenic properties [14]. Low-molecular-weight PAHs (LMW-PAHs), particularly naphthalene (NAP), fluorene (FLU), and phenanthrene (PHE), predominate in mainstream tobacco smoke [18]. Furthermore, tobacco smoke and indoor cooking emissions contribute substantially to LMW-PAH fractions in pulmonary particulate matter (representing 60–75% of total PAHs), whereas atmospheric PM2.5 serves as the primary vector for high-molecular-weight PAHs (HMW-PAHs) [19]. Among the 16 parent PAHs designated as priority pollutants by the US Environmental Protection Agency (US EPA), individual compounds exhibit distinct physical, chemical, and toxicological profiles, ranging from non-carcinogenic to potent mutagenic agents [20].

The route of exposure critically determines the internal dose and tissue distribution of PAHs. Following inhalation, PAHs deposit directly in the respiratory tract, where the lungs serve as both the primary organ of accumulation and a major site for local metabolic processing [20]. The tissue retention of individual PAHs is heavily governed by their physicochemical characteristics, particularly the octanol–water partition coefficient (${\log K}_{ow}$). HMW-PAHs possess higher ${\log K}_{ow}$ values and greater hydrophobicity, driving them to partition preferentially into lipid-rich cellular environments [21,22]. Consequently, organic lipid content plays a central role in governing PAH bioaccumulation and retention within tissue samples [20]. Although HMW-PAHs bind tightly to lipophilic matrices, their biological availability differs from LMW-PAHs, which are more water-soluble and readily bioavailable [20]. Upon biological uptake, PAHs undergo biotransformation, particularly in tissues rich in metabolic phase I and phase II enzymes, with HMW-PAHs generally undergoing enzymatic clearance at faster rates than LMW-PAHs [20,23].

Despite the compelling epidemiological links between environmental pollution, tobacco smoke, and pulmonary disease, direct evidence characterizing internal PAH accumulation within human lung tissue remains limited. Therefore, this study aimed to evaluate and characterize the profile of the 16 US EPA priority PAHs directly in human lung tissue specimens obtained from lung cancer patients and non-cancer control patients in Northern Thailand. Additionally, we sought to evaluate differences in tissue PAH burden between smokers and non-smokers across matched tissue samples. Findings from this investigation provide rare, tissue-level insights into PAH bioaccumulation dynamics, contributing to a better understanding of environmental exposure pathways and informing public health policy in endemic high-exposure regions.

## 2. Materials and Methods

### 2.1. Human Lung Tissue Samples

This study was conducted in accordance with the Declaration of Helsinki and was approved by the Research Ethics Committee of the Faculty of Medicine, Chiang Mai University (Approval No. 414/2023). Prior to surgical intervention and tissue collection, written informed consent was obtained from all participating patients for the research use of their resected biological specimens and associated clinical data. Matched lung cancer tissue samples were obtained from the institutional tissue biobank. Non-cancerous control lung tissues were acquired under identical consent protocols from patients undergoing surgical resection for benign, non-malignant pulmonary conditions. Sample retrieval, processing, and tissue analyses were performed between 1 December 2023, and 30 October 2024. Because no prior studies have evaluated polycyclic aromatic hydrocarbon (PAH) profiles directly in human lung tissue from this high-exposure region, this investigation was designed as an exploratory pilot study (*n* = 15 per group) to establish baseline tissue parameters and preliminary effect sizes. A total of 30 participants were prospectively enrolled, comprising 15 patients with histologically confirmed lung cancer (LC) and 15 control patients without lung cancer (NC). Surgical lung tissue specimens (5 mm^3^ each) were obtained intraoperatively during video-assisted thoracoscopic surgery (VATS) or open thoracotomy. For each patient in the LC group, three matched, paired tissue specimens were harvested from the same resected lung specimen: (i) normal lung tissue from the cancer patient (LC-NT), (ii) pre-cancerous/para-cancerous tissue (LC-PC), and (iii) lung cancer tissue (LC-AC). Para-cancerous tissue was defined as non-tumorous lung parenchymal tissue located within a 2 cm margin from the primary tumor border [24]. This yielded a total of 45 tissue specimens from the LC cohort. For each patient in the NC group, a single non-cancerous normal lung tissue specimen (NC-NT) was collected during surgery for benign pulmonary conditions, yielding 15 control tissue specimens. In total, the study evaluated 60 lung tissue specimens across 30 individual participants (45 specimens from the LC group and 15 from the NC group). Because multiple matched tissue specimens were collected from each lung cancer patient, intra-individual tissue observations were treated as correlated repeated measures and analyzed using Generalized Estimating Equations (GEE) models with appropriate correlation structures.

Histological identification and tissue type demarcation (normal, pre-cancerous, and tumor) were confirmed under the direct guidance of an experienced pulmonary pathologist. Accordingly, three matched tissue specimens were collected from each lung cancer patient (LC-NT, LC-PC, and LC-AC), and a single control tissue specimen (NC-NT) was harvested from each non-cancer participant for quantitative PAH bioaccumulation analysis. Baseline demographic and clinical characteristics—including age, sex, smoking status, cumulative smoking pack-years, comorbidities, Charlson Comorbidity Index (CCI), mask usage, residential air purifier use, and occupational environmental exposures—were comparable between the lung cancer and non-cancer control groups, with no statistically significant differences observed (Table 1).

### 2.2. PAHs Extraction and Analysis

#### Optimization of PAHs Extraction from Lung Tissues

A. Pig lung tissue

The PAH extraction protocol was adapted and modified from a previously established method for PM 2.5-bound PAHs [11]. Due to the limited availability and precious nature of human lung tissue, initial optimization of the extraction procedure was performed using pig lung tissue as a surrogate biological matrix. Sectioned pig lung tissue specimens (average wet weight approximately 50 mg were spiked with 100 µL of a 16-PAH mixed standard solution (40 ng/mL) to achieve a target fortification concentration of 4 ng/mL. Acceptable analytical performance criteria for optimization were defined as a mean recovery rate of 80–120% and a relative standard deviation (RSD) of <15%.

B. Human lung tissue

The optimized extraction conditions established in the surrogate pig lung matrix were subsequently applied to human lung tissue to evaluate method performance and matrix effects in clinical specimens. Approximately 50 mg of wet human lung tissue per sample was processed in two parallel analytical sets: (1) unfortified blank samples to determine baseline endogenous tissue concentrations, and (2) fortified matrix-spiked samples (4 ng/mL 16-PAH standard mixture) to evaluate analytical recovery and precision. Each tissue sample was transferred to a glass extraction tube containing 5 mL of a hexane: dichloromethane (1:1, *v*/*v*) solvent mixture. Ultrasonication-assisted extraction was performed at 10 °C for 45 min. The resulting extract was filtered through a 0.22 µm hydrophobic polytetrafluoroethylene (PTFE) syringe filter. The residual tissue matrix was rinsed twice with hexane, and the combined organic extracts were concentrated under a gentle stream of nitrogen gas to a volume of < 1 mL. Subsequently, 100 µL of an internal standard mixture containing acenaphthene-d_10_ and perylene-_12_ (PER-d_12_) was added to yield final concentrations of 30 ng/mL and 60 ng/mL, respectively. Finally, the extract was reconstituted with the extraction solvent mixture to a total final volume of 1000 µL prior to GC-MS analysis.

### 2.3. PAHs and Cholesterol Analysis by GC-MS

Quantitative profiling of the 16 US EPA priority PAHs was performed using a gas chromatograph coupled with a mass spectrometer (GC-MS; Agilent 7820A GC system coupled to an Agilent 5977E MS detector; Agilent Technologies, Santa Clara, CA, USA). Chromatographic separation was achieved on an Rxi-5Sil MS capillary column (30 m×0.25 mm i.d., 0.25 μm film thickness; Restek, Bellefonte, PA, USA). High-purity helium was used as the carrier gas at a constant flow rate.

The GC oven temperature program was configured as follows: initial temperature held at ${70\;}^{\circ }C$ for 2 min, ramped at ${10\;}^{\circ }C/\min$ to ${190\;}^{\circ }C$, increased at ${8\;}^{\circ }C/\min$ to ${240\;}^{\circ }C$, and finally ramped at ${8\;}^{\circ }C/\min$ to ${290\;}^{\circ }C$, where it was maintained for 8 min (total run time: 32.5 min). The mass spectrometer was operated in electron ionization (EI) mode with data acquisition conducted simultaneously in selective ion monitoring (SIM) mode for target PAH quantification and full-scan mode for non-targeted screening.

The target analyte panel comprised 16 priority PAHs: naphthalene (NAP), acenaphthylene (ACY), acenaphthene (ACE), fluorene (FLU), phenanthrene (PHE), anthracene (ANT), fluoranthene (FLA), pyrene (PYR), benz[a]anthracene (BaA), chrysene (CHR), benzo[b]fluoranthene (BbF), benzo[k]fluoranthene (BkF), benzo[a]pyrene (BaP), indeno [1,2,3-cd]pyrene (IND), dibenz[a,h]anthracene (DbA), and benzo[g,h,i]perylene (BPER).

Endogenous tissue cholesterol was incidentally detected during untargeted full-scan acquisition. Compound identity was confirmed via mass spectral matching against the National Institute of Standards and Technology (NIST) spectral library using MassHunter Software version E.02.02 (molecular ion m/z=386). Because an authentic cholesterol reference standard was not incorporated during initial batch processing, cholesterol levels were evaluated semi-quantitatively. Relative cholesterol abundance was expressed as a percentage, calculated from the ratio of the cholesterol peak area to the internal standard peak area (×100) and normalized to wet tissue weight (mg). Consequently, reported cholesterol values represent a non-targeted, relative assessment rather than absolute tissue concentrations.

### 2.4. Quality Assurance and Quality Control for GC-MS Analysis

The concentrations of individual PAHs were determined using calibration curves constructed from standard solutions spanning a concentration range of 0.4–10 ng/mL. Analyte quantification was performed based on the ratio of the target analyte peak area to that of the corresponding internal standard. The instrumental limits of detection (LODs) for PAHs analysis were determined by analyzing seven replicate GC-MS injections of a 0.4 ng/mL mixed standard solution. The calculated LODs ranged from 0.06 to 0.54 ng/mL, while the limits of quantitation (LOQs) ranged from 0.21 to 1.82 ng/mL. As previously described, the analytical method was initially validated using pig lung tissue as a surrogate biological matrix to establish baseline method performance, successfully achieving mean recovery values of 80−120% with relative standard deviations (RSDs) of <15%. Subsequently, method verification was conducted using human lung tissue to rigorously evaluate matrix-specific effects in clinical specimens. Analytical accuracy and precision were assessed by fortifying human lung tissue samples with a known concentration of the PAH standard mixture (4 ng/mL) and processing them under the identical analytical protocol. Across replicate human tissue analyses (n=6), the mean recoveries for the 16 target PAHs ranged from 69% to 134%, with RSDs ranging from 4% to 26% (Table 2).

### 2.5. Benzo[a]pyrene Equivalents (BaPeq) Calculation

To assess the overall carcinogenic potential of the PAH mixtures detected in human lung tissue samples, total benzo[a]pyrene equivalents (BaPeq) concentrations were calculated (Equation (1)). This toxicity weighting approach accounts for the varying carcinogenic potencies of individual PAH compounds relative to benzo[a]pyrene (BaP), a well-established reference carcinogen. The BaPeq concentration for each sample was calculated using the following formula:

BaPeq = ∑(Ci × TEFi)(1)

where

Ci is concentration of the individual PAH in the sample (ng/g tissue).

TEFi is toxic equivalency factor of the individual PAH relative to BaP (Table 2) [25].

### 2.6. Data Analysis

Baseline demographic and clinical characteristics between the lung cancer (LC) and non-cancer (NC) groups were compared using Fisher’s exact test or Pearson’s chi-square test for categorical variables, and Student’s *t*-test or the Mann–Whitney U (Wilcoxon rank-sum) test for continuous variables, as appropriate based on data distribution normality. To account for intra-individual correlation among paired, repeated tissue measurements harvested from the same patient (LC-NT, LC-PC, and LC-AC), Generalized Estimating Equations (GEE) with an exchangeable correlation structure were employed. Multivariable linear regression and GEE regression models were constructed to evaluate differences in total and individual tissue PAH concentrations across tissue types while adjusting for potential biological and environmental confounders, including age, sex, smoking status, mask usage, and occupational exposure. Adjusted beta coefficients, 95% confidence intervals (95% CI), and adjusted *p*-values were calculated. Exploratory subgroup analyses stratified by smoking status (smokers vs. non-smokers) and cumulative smoking pack-years were conducted to evaluate subgroup-specific bioaccumulation patterns. Non-parametric Spearman rank correlation (r) was used to assess relationships between tissue cholesterol abundance and individual PAH classes. All statistical analyses were performed using Stata/SE software (version 17.0; StataCorp LLC, College Station, TX, USA). A two-tailed *p*-value of < 0.05 was considered statistically significant.

## 3. Results

### 3.1. Profile of Accumulated PAHs and Their Carcinogenic Potency in Human Lung Tissues

PAH concentrations were compared among lung tissues from lung cancer patients (LC-NT, LC-PC, and LC-AC) and non-lung cancer patients (NC-NT) in both non-smoking and smoking groups (see more details in Table S1).

**Figure 1:**
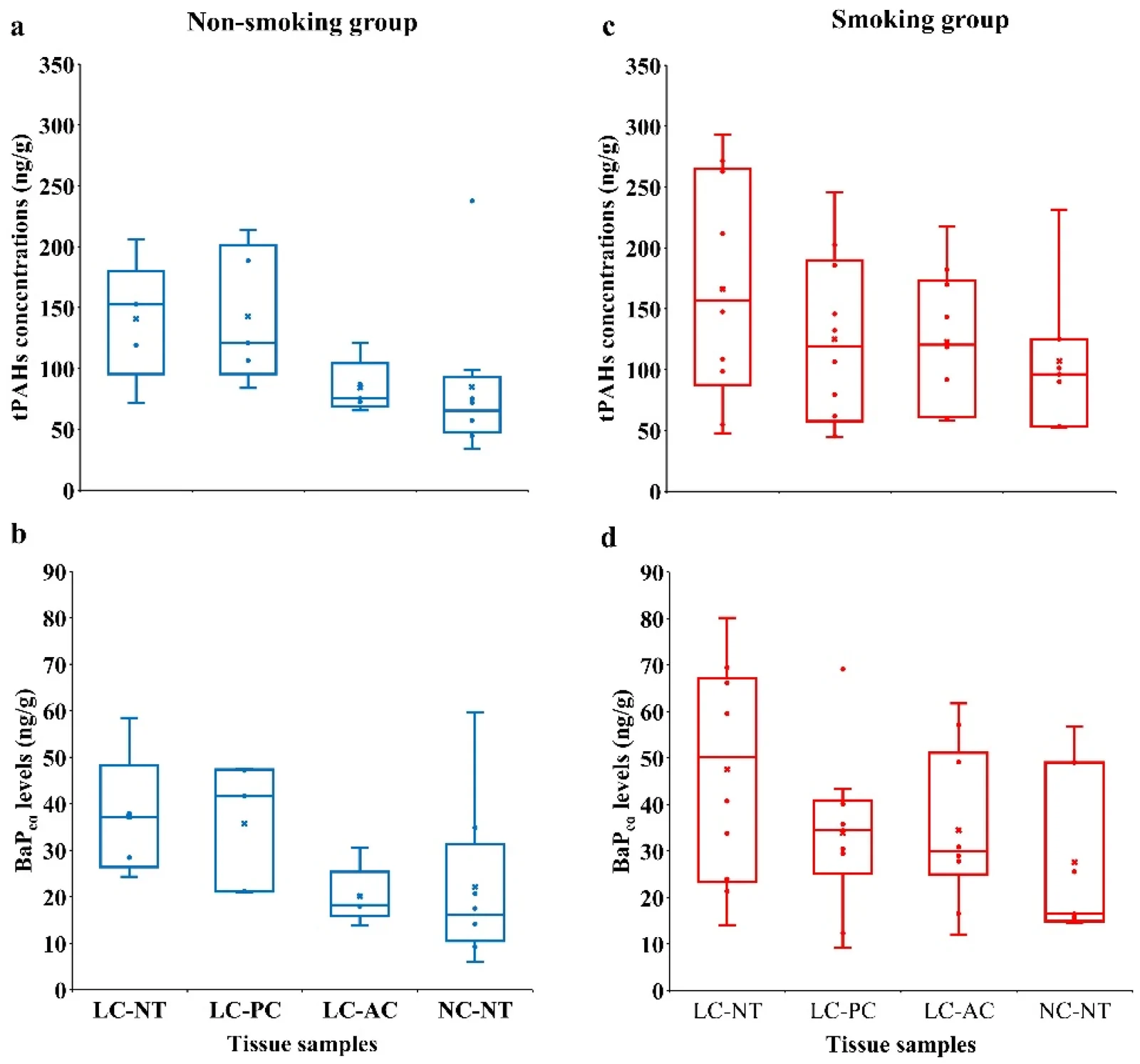
Total PAH concentrations and benzo[a]pyrene equivalent concentrations (BaPeq) in lung tissue samples from the non-smoking group (**a**,**b**) and the smoking group (**c**,**d**). Lung cancer tissues included normal tissue (LC-NT; *n* = 10), para-cancerous tissue (LC-PC; *n* = 10), and lung cancer tissue (LC-AC; *n* = 10) for the non-smoking group, and LC-NT (*n* = 5), LC-PC (*n* = 5), and LC-AC (*n* = 5) for the smoking group. Normal lung tissues from non-lung cancer patients (NC-NT) included *n* = 7 non-smoking samples and *n* = 8 smoking samples.

To evaluate whether PAH accumulation differed across tissue types independently of potential confounders, multivariable linear regression and Generalized Estimating Equations (GEE) models were performed, adjusting for age, sex, smoking status, mask use, and occupation (Table S2). In the overall comparison of lung cancer versus non-cancer lung (NC-NT) tissue, total PAHs were significantly higher in lung cancer tissue, driven primarily by non-carcinogenic PAHs (ncPAHs). Among individual compounds, lung cancer tissue demonstrated significantly elevated levels of BbF, DbA, and BPER, as well as a significant inverse association with IND. In pairwise tissue-type comparisons, normal tissue from lung cancer patients (LC-NT) exhibited significantly higher levels of DbA, BPER, and overall ncPAHs compared with NC-NT controls. Pre-cancerous tissue from lung cancer patients (LC-PC) showed significantly higher accumulations of BbF, DbA, and BPER. Finally, direct comparison of lung cancer tissue (LC-AC) to NC-NT confirmed independently higher levels of BPER. Based on smoking status, tissue samples were categorized into a non-smoking group and a smoking group (the latter encompassing passive smokers, active smokers, and former smokers). In the non-smoking group (Figure 1a,b), higher total accumulated PHA (tPAH) concentrations were observed in tissue samples from lung cancer patients, particularly in LC-PC (142.72 ± 55.60 ng/g) and LC-NT (140.60 ± 49.53 ng/g). These tissue concentrations were approximately 1.6-fold higher than those observed in LC-AC (84.32 ± 22.02 ng/g) and NC-NT controls (84.82 ± 64.79 ng/g). Notably, accumulated tPAH levels in LC-AC and NC-NT were comparable. Evaluated by BaPeq levels, the carcinogenic potency of PAHs was highest in LC-NT, followed by LC-PC, NC-NT, and LC-AC (Figure 1b). However, the ratios of carcinogenic PAHs (cPAHs) to ncPAHs remained similar across all tissue types, ranging from 1.0 to 1.2. Compared with the non-smoking group, both tPAH and BaPeq levels in the smoking group were highest in LC-NT (166.20 ± 90.16 ng/g and 47.56 ± 23.55 ng/g, respectively), whereas concentrations in other tissues types were comparable, ranging from 107 to 125 ng/g for tPAHs and 27.55 to 34.34 ng/g for BaPeq (Figure 1c,d).

The detection frequencies of individual PAHs varied across tissue types, reflecting both compound-specific biological levels and analytical performance characteristics. Naphthalene (NAP), which exhibited the highest limit of quantification (LOQ = 1.82 ng/mL) and the greatest analytical variability (RSD ≈ 26%) during method validation, was not detected in any human lung tissue samples. Similarly, acenaphthene (ACE), which demonstrated the lowest recovery rate during method verification (69 ± 7%), was detected in only two samples: one adenocarcinoma tissue from a non-smoker (LC-AC) and one normal lung tissue specimen from a smoker (LC-NT). Benz[a]anthracene (BaA) and chrysene (CHR) were also detected infrequently, appearing only in isolated sample or specific subgroups, indicating that their concentrations were generally below the limit of detection (LOD) across this cohort. Overall, the sparse detection frequencies of these specific compounds confirm that they contributed minimally to total accumulated PAH concentrations and the calculated carcinogenic potency (BaPeq).

**Figure 2:**
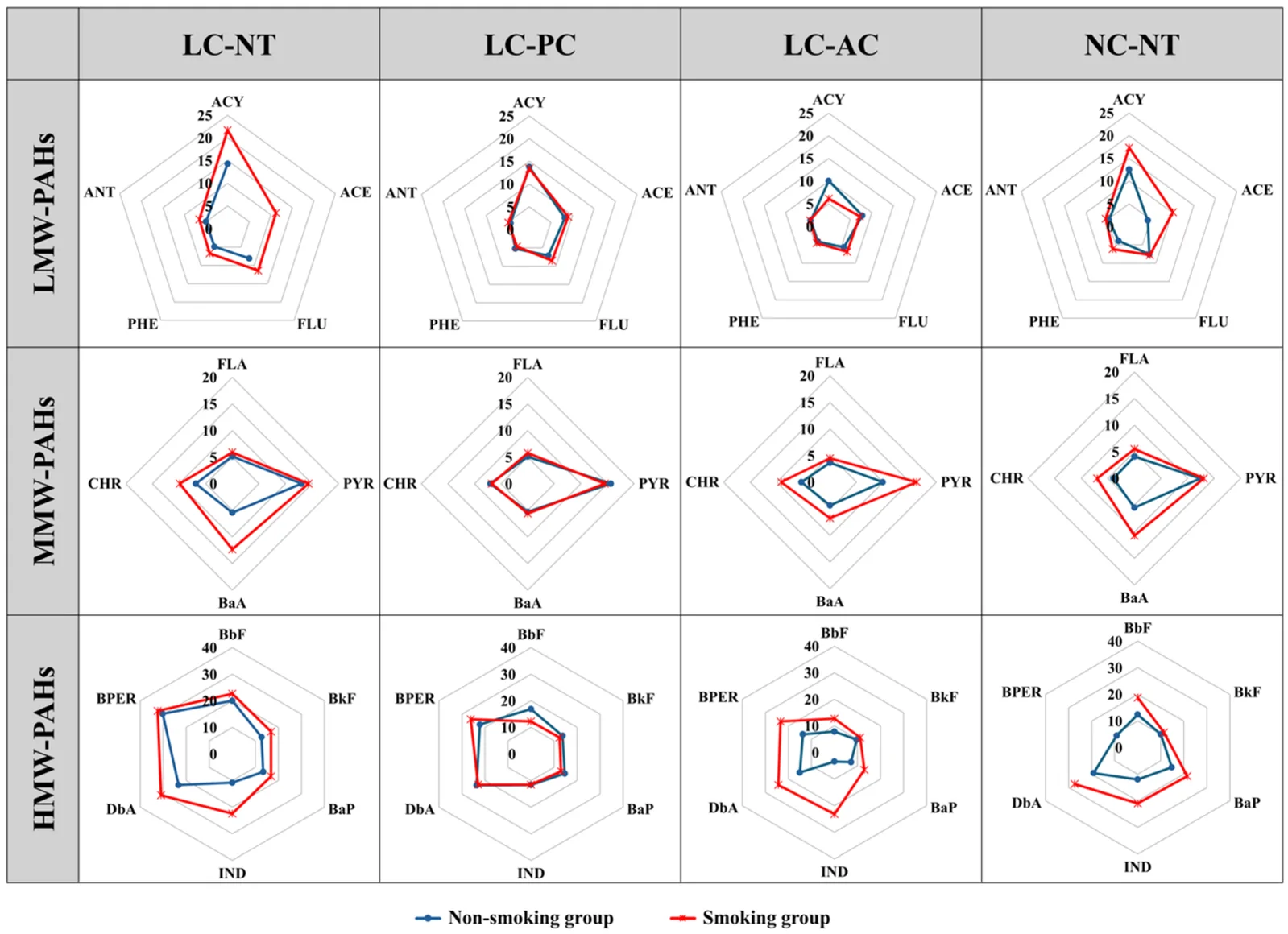
Comparison of individual accumulated PAH concentrations between non-smoking and smoking groups, categorized by molecular weight: low molecular weight (LMW), medium molecular weight (MMW), and high molecular weight (HMW). Data are presented for each tissue sample. The sample groups and sample sizes are the same as those described in Figure 1.

Figure 2 presents a detailed comparison of individual PAH compounds, classified by ring size and molecular weight, between the non-smoking and smoking groups. HMW-PAHs (5–6 rings) represented the most dominant group, comprising over 50% of tPAHs across all analyzed tissue samples.

The association between pack-years of smoking and total PAHs accumulation across three distinct lung tissue types was evaluated, and analyzed using Generalized Linear Models (GLM) with a Poisson family and log link function (Table S3). Overall, no statistically significant association was observed between pack-years and total PAHs accumulation in any of the tissue groups. In non-lung cancer tissue, each additional pack-year was associated with an incidence rate ratio (IRRs) of 0.99 (95% CI: 0.91–1.10, *p* = 0.961). Similarly, in para-cancerous tissue and lung cancer tissue, the estimated IRRs were both 1.01 (95% CI: 0.91–1.11, *p* = 0.915 and 95% CI: 0.92–1.11, *p* = 0.799, respectively). Across all tissue types, the 95% confidence intervals spanned 1.00, indicating that smoking history measured in pack-years was not significantly related to PAH levels in these tissues.

### 3.2. Effect of Cholesterol on the Accumulation of PAHs

**Figure 3:**
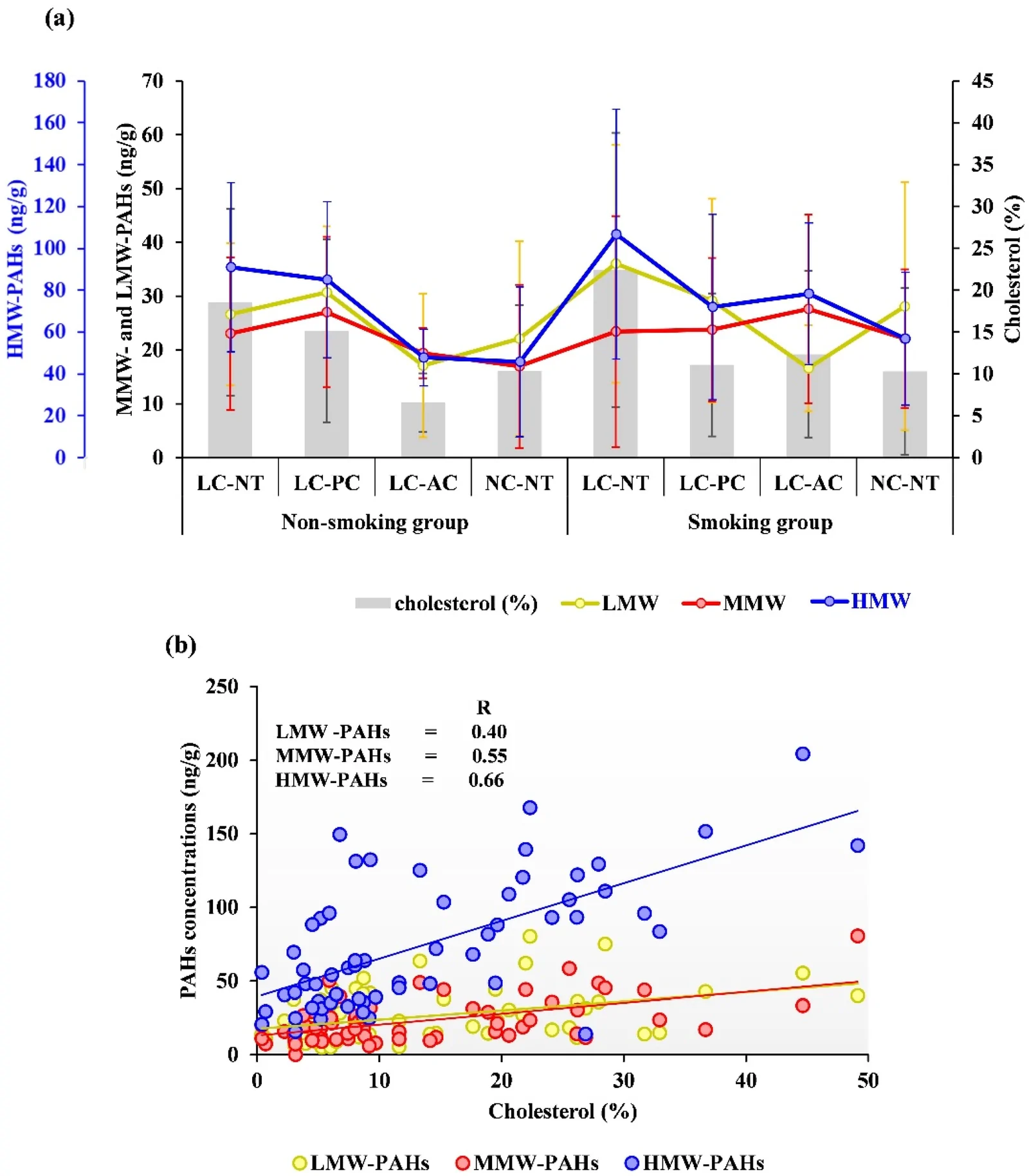
Association between accumulated PAHs, categorized by molecular weight, and the relative percentage of cholesterol in tissue sample: (**a**) average concentrations of PAHs and the relative percentage of cholesterol in each tissue (LC-NT, LC-PC, and LC-AC (*n* = 5 smokers and *n* = 10 non-smokers for each tissue type); NC-NT (*n* = 8 smokers and *n* = 7 non-smokers); and (**b**) correlations between PAH concentrations and cholesterol content (*n* = 60).

Driven by the lipophilic nature of tissue matrices and the physicochemical properties of organic pollutants, endogenous cholesterol was incidentally detected during GC-MS analysis of target PAHs. In this study, tissue cholesterol content is reported as relative percentage abundance due to the absence of an authentic reference standard. Notably, the lowest concentrations of accumulated PAHs were observed in LC-AC tissue specimens, representing malignant cancerous tissue. As shown in Figure 3a, relative cholesterol abundance was significantly lower in LC-AC compared to all other tissue samples in the non-smoking cohort. Conversely, within the smoking cohort, the highest relative cholesterol abundance coincided with the highest total accumulated PAH concentrations, both observed in LC-NT specimens. Evaluation of the relationship between tissue PAH concentrations and relative cholesterol levels demonstrated a statistically significant positive correlation across all compound classes (Figure 3b). This positive association was strongest for high-molecular-weight PAHs (HMW-PAHs; r=0.66,p<0.001), followed by medium-molecular-weight PAHs (MMW-PAHs; r=0.55,p<0.001) and low-molecular-weight PAHs (LMW-PAHs; r=0.40,p<0.001).

### 3.3. Relationship Between the Carcinogenic Potency of PAHs (BaPeq) and Tobacco Markers (LMW-PAHs)

**Figure 4:**
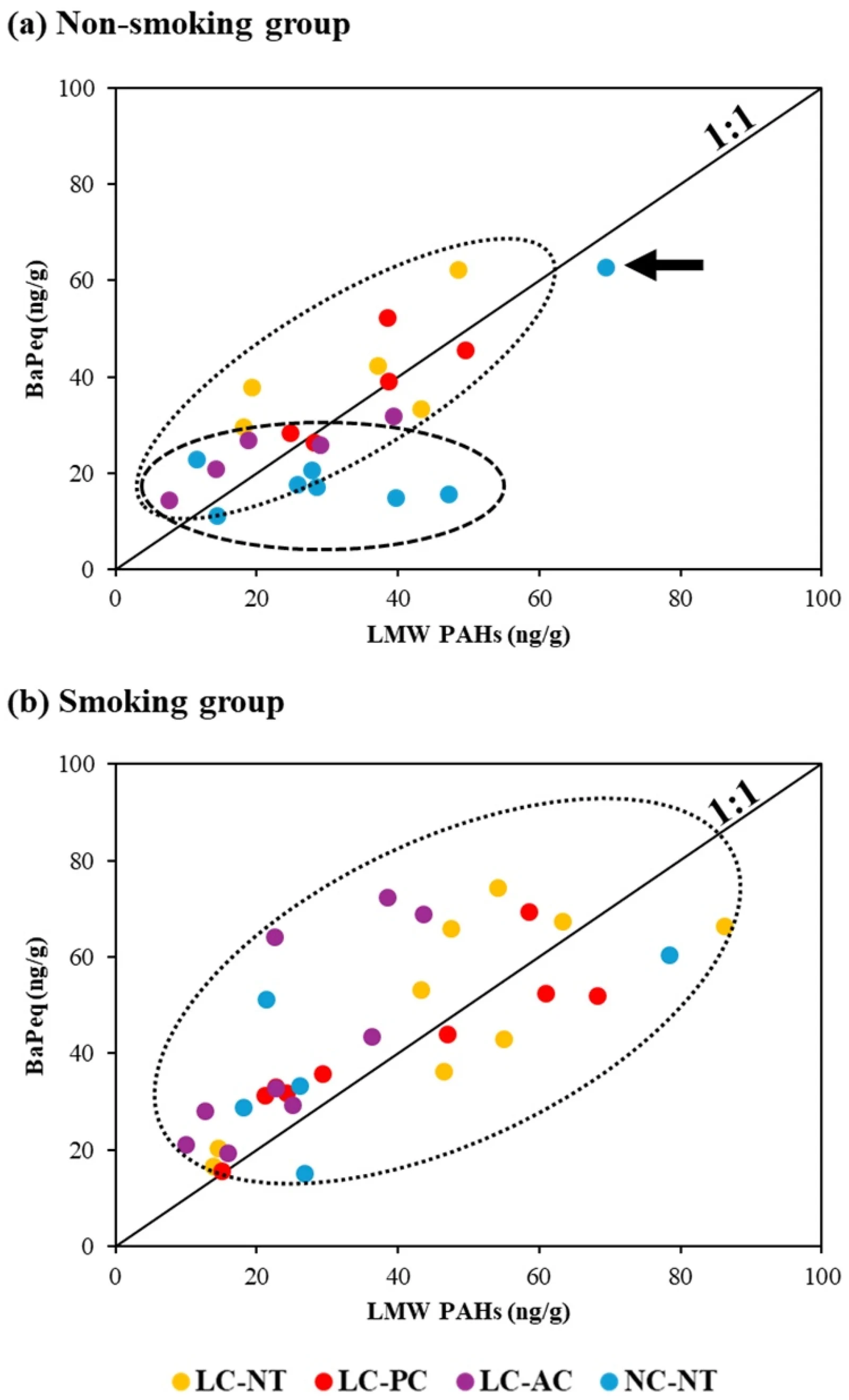
Ratios and correlations between BaPeq and low molecular weight (LMW) PAHs (tobacco markers) in each tissue type for (**a**) non-smoking group (*n*= 10 for each lung cancer tissue samples (LC) and *n* = 7 for NC-NT) and (**b**) smoking group (*n*= 5 for each lung cancer tissue samples (LC) and *n* = 8 for NC-NT). Dotted circles indicate groups with a 1:1 ratio, a dashed circle indicates groups with constant BaPeq values, and an arrow indicates a sample from patient who worked in the plastic recycling industry.

**Figure 5:**
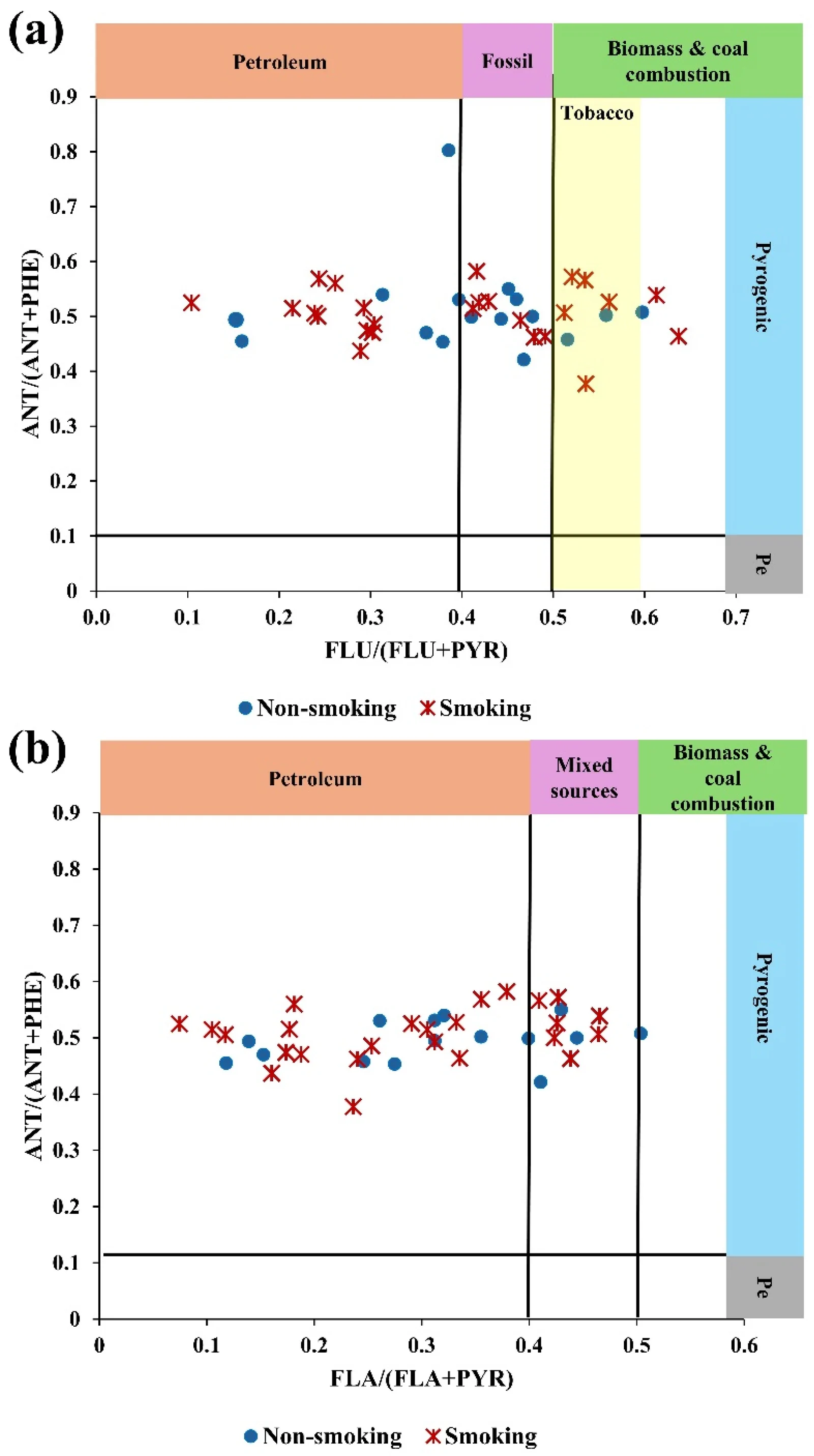
Potential sources of accumulated PAHs in lung tissues of non-smoking (*n* = 37) and smoking groups (*n* = 23), based on diagnostic ratios: (**a**) ANT/(ANT+PHE) versus FLU/(FLU+PYR) and (**b**) ANT/(ANT+PHE) versus FLA/(FLA+PYR). “Pe” indicates petrogenic sources.

In this study, the relationship between BaPeq and LMW-PAHs, as well as their corresponding concentration ratios, was analyzed across all tissue samples. As shown in Figure 4, the BaPeq-to-LMW-PAHs ratio remained relatively consistent across most tissue types in both non-smoking and smoking patients; however, a distinct divergence emerged in NC-NT tissues among non-smoking individuals. In non-smokers (Figure 4a), BaPeq concentrations in NC-NT tissues remained stable (dashed circle), whereas BaPeq in other tissue types increased proportionally alongside rising LMW-PAH concentrations (dotted circle). In contrast, this distinct pattern was not observed in smokers (Figure 4b). Nevertheless, substantial carcinogenic potency was observed across samples, primarily driven by the accumulation of highly carcinogenic species such as BaP and DbA, which possess the highest toxic equivalency factors (TEF). Notably, three LC-AC specimens from smokers exhibited markedly elevated BaPeq values despite demonstrating LMW-PAH concentrations comparable to other tissue specimens (Figure 4b). Figure 5 presents scatter plots of the diagnostic ratios ANT/(ANT+PHE) versus FLU/(FLU+PYR) and FLA/(FLA+PYR) for both non-smoking and smoking groups. These specific diagnostic ratios were selected for source apportionment analysis because PHE was detected in 78% of the samples, while the remaining four target PAHs (FLU, ANT, FLA and PRY) were consistently detected in over 95% of the samples.

## 4. Discussion

The association between tPAHs burden and human lung carcinogenesis remains a subject of intense investigation. We hypothesize that long-term exposure to ambient PM2.5 and inhaled combustion emissions promotes the direct accumulation of tPAHs within human lung tissue, contribution to a pro-carcinogenic microenvironment. In this study, we evaluated paired lung tissue specimens from both lung cancer patients and non–lung cancer patients, observing significantly higher accumulated tPAHs concentrations in the lung cancer group. Our findings indicate that accumulated tPAH levels in LC-AC and NC-NT specimens from non-smoking individuals are comparable to tissue PAH concentrations previously reported in pulmonary cancer patients residing in urban areas of Romania (approximately 70 ng/g), while being markedly higher than those observed in rural Romanian populations (approximately 30 ng/g) [26]. Furthermore, non-cancer patient with normal lung tissue in our Northern Thailand cohort demonstrated substantially higher concentrations of individual target PAHs compared to cancer-free lung tissue from Caucasian and African-American autopsy donors in North America [27] as well as autopsy donors in Spain [22] (Table S4).

Our study found that HMW-PAHs (5–6 rings) represented the predominant class in our cohort, accounting for more than 50% of tPAHs across all analyzed tissue samples. This profile aligns with the physicochemical characteristics of HMW-PAHs, which exhibit high octanol–water partition coefficients (${\log K}_{ow}$), lower volatility and a strong thermodynamic propensity to bind to airborne particulate matter (PM). Their tissue predominance mirrors atmospheric environmental distributions in Southeast Asia, particularly Northern Thailand, where seasonal smoke haze episodes during the dry season (March–April) driven by extensive open biomass burning generate severe air pollution [11,28,29]. During these peak pollution periods, atmospheric PM are heavily enriched with HMW-PAHs (especially 5- and 6-ring compounds), displaying strong positive correlations with ambient PM2.5 concentrations (r = 0.6–0.7) [11]. Insian et al. [12] previously reported that BbF was the single most abundant PAH compound across all particle size fractions in Chiang Mai, contributing 23–24% of total priority PAHs, and attributed its primary origin to open biomass combustion [30]. Conversely, during non-haze periods, IND, BPER, and DbA predominate, reflecting vehicular traffic emissions [31,32,33]. Due to their high marked lipophilicity, inhaled HMW-PAHs preferentially partition into and persist within lipid-rich biological compartments. Furthermore, the overall tPAHs level observed in our lung cancer tissue are comparable to concentrations reported in lung cancer specimens from the Beijing-Tianjin-Hebei region of China (approximately 140 ng/g)-a heavily industrial urban center characterized by severe chronic air pollution (Table S4) [19].

Accumulated pulmonary PAH concentrations were generally higher in the smoking group than in the non-smoking group, characterized by elevated levels of both LMW-PAHs and HMW-PAHs. Beyond the baseline presence of HMW-PAHs, LMW-PAHs are recognized as characteristic “fingerprint” compounds of tobacco smoke [19]. We also observed a smoking-related increase in ncPAHs such as FLA and PYR, which tend to accumulate in parenchymal tissue with age [27]. However, LC-PC tissues exhibited similar PAH concentrations across both smoking and non-smoking groups, suggesting that local tissue architecture or microenvironmental factors beyond active smoking influence accumulation kinetics in pre-cancerous tissue zone. Nevertheless, tobacco smoking remains a major contributor that amplifies tissue PAH burden and accelerates carcinogenic risk.

Evaluating the relationship between tissue PAH concentrations and relative cholesterol abundance revealed a statistically significant positive correlation across all compound classes, with the strongest correlation observed for HMW-PAHs, followed by MMW-PAHs and LMW-PAHs. This biological pattern is supported by in vivo tissue distribution studies demonstrating that PAHs and their lipophilic derivatives exhibit low tissue mobility and high retention in lipid matrices, making the lungs a primary target organ following inhalation exposure [20]. Similar, human autopsy studies have recorded the highest relative PAH accumulation in lipid-dense organ systems, including the liver and brain [22]. These findings clarify the differential accumulation patterns observed across tissue compartments and suggest that endogenous tissue lipid content serves as a key physicochemical “depot capacity” governing PAH retention. Furthermore, these observations intersect with the concept of lipid metabolic reprogramming—an established hallmark of cancer reflecting adaptations that malignant cells undergo to meet bioenergetic and biosynthetic demands [2]. Within the tumor microenvironment, cancer cells frequently alter membrane phospholipid composition and upregulate de novo lipogenesis to support rapid proliferation and altered cell signaling [2]. The lower parent PAH concentrations observed in established adenocarcinoma tissues (LC-AC) compared to adjacent normal tissues (LC-NT) may therefore reflect the altered metabolic state of malignant cells, which can promote enhanced enzymatic biotransformation, cellular efflux, or tissue redistribution [2,3]. Crucially, reduced parent PAH levels in fully developed tumors do not negate their causative role in pathogenesis: PAHs act as pro-carcinogens that primarily initiate tumor formation during the initiation phase via CYP-mediated metabolic activation and subsequent formation of covalent DNA adducts [4,7]. Thus, while parent PAH levels in lung tissue largely reflect passive partitioning into a lipophilic tissue depot, malignant transformation alters this retention capacity through metabolic adaptation and microenvironmental remodeling. Tobacco smoke is a major cause of lung cancer worldwide [7,9]. Chemical analysis of tobacco smoke emissions indicates that LMW-PAHs constitute the predominant fraction, accounting for up to 82% of total emitted PAHs [18]. This aligns with extensive literature establishing LMW-PAHs as the primary PAH species in mainstream tobacco smoke due to their high volatility and preferential formation in the gas phase during cigarette combustion [19,34,35]. This is attributed to the fact that LMW-PAHs exist primarily in the gas phase and are readily formed during the combustion of tobacco products [18]. Consequently, LMW-PAHs serve as sensitive bio-markers for tobacco exposure [19]. Although individual LMW-PAHs are not formally classified as Group 1 human carcinogens by the International Agency for Research on Cancer (IARC) [36], they can act as co-carcinogens or tumor promoters by enhancing the potency of other carcinogenic agents [37,38]. Therefore, substantial accumulation of LMW-PAHs from tobacco exposure contributes meaningfully to cumulative toxic burden and overall toxic equivalency (BaPeq). PAH diagnostic ratios are widely applied in environmental sciences to evaluate pollution origins across atmospheric, soil, aquatic, and biological matrices [39,40,41,42,43]. In this study, PAHs diagnostic ratios were determined in lung tissue to provide preliminary insights into potential PAH exposure sources. An ANT/(ANT+PHE) diagnostic ratio has been widely used to distinguish between petrogenic and pyrogenic PAH sources, with values < 0.1 generally indicating petrogenic sources and values > 0.1 indicating pyrogenic sources [40,42]. In this study, the average ANT/(ANT+PHE) ratio was 0.51 ± 0.06, which is consistent with a predominance of pyrogenic PAH exposure in both groups. [44]. However, because these diagnostic ratios were originally developed for environmental matrices, their interpretation in biological tissues should be considered preliminary, as differential uptake, metabolism, and elimination may alter the relative abundances of individual PAHs after accumulation in lung tissue. Nevertheless, the observed ANT/(ANT+PHE) ratios are comparable to those previously reported for ambient PM2.5 collected in Chiang Mai, Thailand, during the 2017–2018 smoke haze episodes, where values ranged from 0.14 to 0.36 and were likewise associated with pyrogenic emission sources [11]. The FLU/(FLU+PRY) diagnostic ratio was primarily associated with three main sources: petroleum emissions (<0.4), fossil fuel emissions (0.4–0.5), and biomass and coal combustion (>0.5) [43,45]. Additionally, a ratio in the range of 0.5–0.6 has been linked to tobacco smoking [39]. In the present study, FLU/(FLU+PYR) ratios ranged from 0.1 to 0.6 in both the non-smoking and smoking groups, suggesting exposure to multiple PAH sources. Higher ratios observed in some individuals may also be consistent with exposure to tobacco smoke, particularly among smokers, whereas exposure among non-smokers may reflect secondhand smoke. FLA/(FLA+PYR) ratios less than 0.5 indicate petroleum emissions, while values in the range of 0.5–0.6 are associated with mixed sources (either petroleum or combustion). Additionally, values greater than 0.6 represent biomass and coal combustion sources [19]. In this study, the average FLA/(FLA+PYR) ratio was 0.31 ± 0.12, with values ranging from 0.07 to 0.50, suggesting that petroleum emissions and mixed combustion sources were the predominant contributors. These observations are generally consistent with previous studies reporting that ambient fine particulate matter in urban environments is influenced by a combination of traffic-related emissions and combustion sources [46]. However, because the diagnostic ratios were determined from lung tissue rather than environmental samples, these findings should be interpreted as preliminary evidence of potential exposure sources rather than definitive source attribution.

This study has several limitations that warrant consideration. First, as an exploratory pilot investigation, the overall sample size was modest (*n* = 30), leading to small numbers in specific subgroups after stratification by smoking history and tissue type. The relatively small number of participants (15 lung cancer patients and 15 non-lung cancer patients) may limit the generalizability of the results to the broader population. Although the repeated-measures design, in which three matched tissue types were obtained from each lung cancer patient, enhanced the efficiency of within-subject comparisons and reduced inter-individual variability, the modest sample size may have limited the ability to detect subtle differences among tissue types or individual PAH compounds. Therefore, non-significant findings should be interpreted with caution. Nevertheless, statistically significant differences in total PAH concentrations and BaPeq between smokers and non-smokers support the robustness of the observed associations within this cohort. Indeed, post hoc power for subgroup comparisons (18.1%) confirms that these secondary analyses are underpowered. Therefore, observed trends regarding smoking status must be interpreted with caution as preliminary pilot data aimed at guiding future larger multi-center cohorts; moreover, this study provides rare, direct evidence of PAH accumulation in human lung tissues from Northern Thailand, a region with a high burden of lung cancer and recurrent exposure to biomass burning. These findings fill an important knowledge gap and establish a valuable foundation for future studies with larger, multi-center cohorts to validate the findings and further investigate the relationship between PAH accumulation and lung carcinogenesis. Second, this study is that only the 16 priority parent PAHs were quantified in lung tissues, whereas hydroxylated PAH metabolites were not evaluated. Consequently, the measured PAH concentrations represent tissue accumulation rather than the biologically effective dose. Furthermore, inter-individual variability in the activity of PAH-metabolizing enzymes, including CYP1A1, CYP1B1, GSTM1, and GSTT1, may influence PAH bioactivation, detoxification, and tissue accumulation, thereby affecting individual susceptibility to carcinogenesis [1]. Future studies integrating PAH metabolites and genetic polymorphisms of metabolic enzymes would provide a more comprehensive assessment of PAH exposure and its role in lung cancer development. Despite these limitations, the findings underscore the complex interplay between environmental exposure, per sonal habits, and biological factors in influencing PAH accumulation and its associated health risks. They highlight the multifactorial nature of PAH bioaccumulation and the critical importance of mitigating both environmental and behavioral risk factors. Third, in this study, the cohort was predominantly composed of adenocarcinoma cases. This reflects contemporary epidemiological trends in northern Thailand and globally, where adenocarcinoma is the dominant histological subtype of non-small cell lung cancer (NSCLC), representing 68.48% compared to 14.86% for squamous cell carcinoma in our data [47]; therefore, our analysis was restricted to adenocarcinoma based on tissue availability within the biobank. Given that squamous cell carcinoma may exhibit a strong association with PAH exposure in smoking-related lung cancers, our findings cannot be generalized to all NSCLC. Future studies specifically targeting squamous cell carcinoma cohorts are warranted to elucidate these relationships further.

## 5. Conclusions

This study provides rare, direct baseline evidence characterizing the bioaccumulation dynamics and carcinogenic profiles of PAHs across matched tissue compartments in human lungs. HMW-PAHs represented the predominant class, accounting for over 50% of tPAHs across all analyzed tissue specimens—a pattern consistent with regional ambient fine particulate matter PM2.5 profiles in Northern Thailand. Comparative analyses demonstrated elevated concentrations of both LMW-PAHs and HMW-PAHs in the lung tissues of smokers relative to non-smokers. Furthermore, the relationship between carcinogenic potency (${\mathrm{BaP}}_{\mathrm{eq}}$) and LMW-PAH accumulation in smokers displayed a 1:1 proportional trend between cancerous and non-cancerous tissue compartments—a pattern distinct from that observed in normal lung tissues of non-smokers. This finding suggests that tobacco smoking facilitates the systemic bioaccumulation of PAHs in non-malignant pulmonary parenchyma to levels comparable with those observed in tumor tissues, potentially heightening tissue susceptibility to malignant transformation.

Additionally, a statistically significant positive correlation between relative tissue cholesterol abundance and PAH concentrations—particularly for HMW-PAHs—indicates that endogenous lipid content serves as a key physicochemical determinant of tissue “depot capacity” for lipophilic carcinogens. Source apportionment analysis identified exposure contributions from multiple sources, including biomass burning, traffic emissions, and tobacco smoke. While these findings offer critical tissue-level insights, the modest cohort size warrants validation in larger, prospective multi-center cohorts. Moreover, the precise cellular and molecular mechanisms governing lipid-mediated PAH retention and intra-tumoral metabolic clearance require further functional investigation. Overall, our findings underscore the multifactorial nature of pulmonary PAH bioaccumulation—driven by a complex interplay between ambient air pollution, behavioral exposures, and biological tissue characteristics—and highlight the urgent necessity of mitigating both environmental pollution and personal risk factors.

## Figures and Tables

**Table 1:** Patient characteristics between lung cancer and non-lung cancer group.

Variables	Lung Cancer Group (*n* = 15)	Non-Lung Cancer Group (*n* = 15)	*p*-Value
Age (years), Mean ± SD	66.35 ± 8.86	65.52 ± 8.42	0.794
Male, n (%)	7 (46.67)	7 (46.67)	1.000
Smoking status, n (%)			0.093
Non-smoker	5 (33.33)	8 (53.33)	
Passive smoker	0	2 (13.33)	
Active smoker	9 (60.00)	3 (20.00)	
Ex-smoker	1 (6.67)	2 (13.33)	
Pack-year, Median (IQR)	10 (5.9–30)	5 (4–8.75	0.194
Insurance type, n (%)			0.573
UCS	9 (60.00)	6 (40.00)	
CSMBS	6 (40.00)	7 (46.67)	
SSS	0	1 (6.67)	
others	0	1 (6.67)	
Comorbid disease, n (%)	14 (93.33)	15 (100.00)	1.000
Hypertension	9 (60.00)	4 (26.67)	0.139
Chronic lung disease	6 (40.00)	2 (13.33)	0.215
Diabetes Mellitus	1 (6.67)	3 (20.00)	0.598
Chlarson comorbidity index, Median (IQR)	3 (3–4)	3 (2–7)	0.401
Mask use, n (%)	11 (73.33)	14 (93.33)	0.330
Frequent mask use in each day			0.525
Not often	3 (20.00)	2 (13.33)	
Often	4 (26.67)	7 (46.67)	
All time outside the house	4 (26.67)	5 (33.33)	
unknown	4 (26.67)	1 (6.67)	
Timing for mask use (years), Median (IQR)	4 (4–5)	4 (4–5)	0.572
Air purifier at home, n (%)	6 (40.00)	5 (33.33)	1.000
Occupation, n (%)			0.424
Outdoor	10 (66.67)	6 (40.00)	
Indoor with air purifier	3 (20.00)	5 (33.33)	
Indoor without air purifier	2 (13.33)	4 (26.67)	

CSMBS, Civil Servant Medical Benefit Scheme; SSS, Social Security Scheme; UCS, Universal Coverage Scheme; IQR, Interquartile range.

**Table 2:** Characteristics of 16 PAHs, limit of Detection (LOD), limit of quantitation (LOQ), percentage extraction recovery of 16 PAHs from spike method, and toxic equivalent factors of each PAHs.

PAHs Compounds	Molecular Formula	Molecular Weight	Ring Number	Carcinogenic Group *	TEF	Quantification Ion (*m*/*z*)	LOD (ng/mL) (*n* = 7)	LOQ (ng/mL) (*n* = 7)	Recovery (%) (*n* = 6)
Naphthalene (NaP)	C_10_H_8_	128.16	2		0.001	128	0.54	1.82	134 ± 35
Acenaphthylene (ACY)	C_12_H_8_	152.20	3		0.001	152	0.43	1.42	110 ± 12
Acenaphthene (ACE)	C_12_H_10_	154.21	3		0.001	154	0.34	1.14	69 ± 7
Flourene (FLU)	C_13_H_10_	166.20	3		0.001	166	0.09	0.30	82 ± 8
Phenanthrene (PHE)	C_14_H_10_	178.20	3		0.001	178	0.18	0.60	81 ± 5
Anthracene (ANT)	C_14_H_10_	178.20	3		0.01	178	0.15	0.49	98 ± 14
fluoranthene (FLA)	C_16_H_10_	202.26	4		0.001	202	0.11	0.36	89 ± 12
Pyrene (PYR)	C_16_H_10_	202.30	4		0.001	202	0.08	0.26	96 ± 10
Benzo[a]anthracene (BaA)	C_8_H_12_	228.29	4	2B	0.1	228	0.08	0.28	99 ± 5
Chrysene (CHR)	C_8_H_12_	228.30	4	2B	0.01	228	0.06	0.21	104 ± 5
Benzo[b]fluoranthene (BbF)	C_20_H_12_	252.30	5	2B	0.1	252	0.20	0.68	91 ± 11
Benzo[k]fluoranthene (BkF)	C_20_H_12_	252.30	5	2B	0.1	252	0.19	0.64	87 ± 18
Benzo[a]pyrene (BaP)	C_20_H_12_	252.30	5	1	1	252	0.38	1.27	84 ± 12
Indeno[1,2,3-cd]pyrene (IND)	C_22_H_12_	276.30	6	2B	0.01	276	0.18	0.60	81 ± 13
Dibenzo[a,h]anthracene (DBA)	C_22_H_14_	278.35	5	2A	1	278	0.29	0.96	91 ± 13
Benzo[ghi]perylene (BPER)	C_22_H_12_	276.34	6		0.01	276	0.15	0.51	77 ± 7

* International Agency for Research on Cancer (IARC): carcinogenic to humans (Group 1), probably carcinogenic to humans (Group 2A), and possibly carcinogenic to humans (Group 2B).

## Data Availability

All data supporting the findings of this study are available within the paper and its Supplementary Information.

## Supplementary Materials

The following supporting information can be downloaded at https://www.mdpi.com/article/10.3390/toxics14080702/s1. Table S1: Average accumulated PAHs and BaPeq levels in human lung tissues from non-smoking and smoking groups; Table S2: Beta-coefficients and 95% confidence interval of Polycyclic aromatic hydrocarbons (PAHs) among non-cancer lung tissue (NC-NT), normal tissue from lung cancer patients (LC-NT), pre-cancerous tissues from lung cancer patients (LC-PC), and lung cancer tissue (LC-AC), adjusted by age, sex, smoking status, mask use, and occupation; Table S3: Association between Pack-year and total PAHs accumulation in each tissue in smoker patients analyzed by Generalized Linear Model (GLM) with a Poisson family and log link function; Table S4: Comparison of accumulated pulmonary PAHs (mean ± SD) between this study and the previous literature [19,22,26,27].

## Abbreviations

The following abbreviations are used in this manuscript:

PAHs	Polycyclic Aromatic Hydrocarbons
ASR	Annual Age-Standardized Rates
PM	Particulate Matter
BTEX	Benzene-Toluene-Ethylbenzene-Xylene
LMW-PAHs	Low Molecular Weight Polycyclic Aromatic Hydrocarbons
HMW-PAHs	High Molecular Weight Polycyclic Aromatic Hydrocarbons
NAP	Naphthalene
ACY	Acenaphthylene
ACE	Acenaphthene
FLU	Fluorene
PHE	Phenanthrene
ANT	Anthracene
FLA	Fluoranthene
PYR	Pyrene
BaA	Benz[a]anthracene
CHR	Chrysene
BbF	Benzo[b]fluoranthene
BkF	Benzo[k]fluoranthene
BaP	Benzo[a]pyrene
BaP_eq_	Benzo[a]pyrene equivalents
IND	Indeno[1,2,3-cd]pyrene
DbA	Dibenz[a,h]anthracene
BPER	Benzo[g,h,i]perylene
tPAHs	Total Polycyclic Aromatic Hydrocarbons
cPAHs	Carcinogenic polycyclic Aromatic Hydrocarbons
ncPAHs	Non-carcinogenic Polycyclic Aromatic Hydrocarbons
US EPA	United States Environmental Protection Agency
VATS	Video-Assisted Thoracic Surgery
LC	Lung Cancer
LC-NT	Lung Cancer-Normal Tissue
LC-PC	Lung Cancer-Para-Cancerous
LC-AC	Lung Cancer-Adenocarcinoma
IQR	Interquartile range
UCS	Universal Coverage Scheme
CSMBS	Civil Servant Medical Benefit Scheme
SSS	Social Security Scheme
RSD	Relative Standard Deviation
PTFE	Polytetrafluoroethylene
ACE-d10	Acenaphthene-d10
PER-d12	Perylene-d12
GC-MS	Chromatography-mass Spectrometry
SIM	Selective Ion Monitoring
LOD	Limits of Detection
LOQ	Limits of Quantitation
TEF	Toxic Equivalency Factor
IARC	International Agency for Research on Cancer
GEE	Generalized Estimating Equation
