# Supplementary material for: Comparative Carcinogenic Risk Assessment of Polycyclic Aromatic Hydrocarbons Accumulation in Normal and Cancerous Lung Tissues of Patients from Northern Thailand
Source: Toxics. 2026 Aug 9;14(8):702. doi: 10.3390/toxics14080702 (PMC13517832; doi:10.3390/toxics14080702)
Supplement: Supplementary file 1 [file toxics-14-00702-s001.zip › toxics-4448520-supplementary.pdf]

Supplementary material

Table S1. Average accumulated PAHs and BaPeq levels in human lung tissues from non-smoking and smoking groups.

PAHs	PAHs concentrations (ng/g)							
	Non-smoking				Smoking			
	LC-NT (n = 5)	LC-PC (n = 5)	LC-AC (n=5)	NC-NT (n=8)	LC-NT (n = 10)	LC-PC (n=10)	LC-AC (n=10)	NC-NT (n = 7)
ACY	14.3 ± 4.38	13.77 ± 2.38	10.08 ± 3.61	12.57 ± 9.46	21.69 ± 10.71	13.41 ± 7.6	6.14 ± 1.41	17.35 ± 6.25
ACE	ND	8.25 ± 1.33	7.79	4.32 ± 0.52	11.22	9.03 ± 6.57	7.19 ± 1.95	10.12 ± 5
FLU	8.11 ± 2.87	7.14 ± 2.36	5.68 ± 2.37	7.6 ± 7.7	11.44 ± 5.91	8.52 ± 4.63	6.89 ± 3.55	7.79 ± 3.74
PHE	4.91 ± 1.38	5.24 ± 1.66	4.07 ± 0.15	3.94 ± 2.09	6.75 ± 2.06	4.65 ± 2.09	4.49 ± 2.26	6.17 ± 4.34
ANT	5.02 ± 1.2	4.34 ± 0.49	4.19 ± 1.08	4.69 ± 2.17	6.56 ± 2.1	4.84 ± 2.32	4.36 ± 1.75	5.46 ± 2.17
FLA	5.12 ± 0.71	5.08 ± 0.66	3.65 ± 0.74	4.1 ± 1.76	5.88 ± 1.89	5.76 ± 3.28	4.51 ± 1.73	5.53 ± 1.9
PYR	13.01 ± 8.9	15.61 ± 12.01	9.91 ± 4.15	12.37 ± 10.21	14.33 ± 13.73	14.51 ± 8.06	16.31 ± 12.75	13.04 ± 8.51
<u>BaA</u>	5.45 ± 1.18	5.3 ± 0.04	4.36 ± 1.23	5.49 ± 3.32	12.33	5.61 ± 2.74	6.75 ± 2.95	10.77
<u>CHR</u>	6.84 ± 0.86	7.01 ± 0.78	5.37 ± 1.21	3.94	9.86 ± 0.14	6.76 ± 3.5	9.18 ± 4	7 ± 0.31
<u>BbF</u>	20.01 ± 4.33	16.9 ± 11.19	7.9 ± 4.1	12.42 ± 6.57	22.7 ± 18.69	12.26 ± 6.27	12.77 ± 5.76	18.76 ± 3.07
<u>BkF</u>	12.73 ± 1.1	13.72 ± 5.24	9.68 ± 2.83	10 ± 8.2	16.8 ± 7.05	12.4 ± 7.78	11.23 ± 4.95	11.38 ± 5.66
<u>BaP</u>	13.42 ± 9.64	14.67 ± 4.94	7.25 ± 4.28	14.8 ± 3.1	16.8 ± 7.19	13.04 ± 5.64	13.04 ± 6.59	21.48 ± 17.85
<u>IND</u>	10.79 ± 0.99	11.65 ± 6.67	3.4 ± 0.9	11.97 ± 9.63	22.39 ± 13.02	11.5 ± 11.19	23.13 ± 17.07	20.95 ± 14.31
<u>DbA</u>	23.34 ± 3.26	23.6 ± 6.52	15.01 ± 3.99	19.12 ± 13.78	30.93 ± 15.36	23 ± 11.19	24.36 ± 11.31	27.39 ± 19.18
BPER	30.22 ± 8.09	22.16 ± 5.09	13.72 ± 3.19	9.09 ± 1.53	32.34 ± 11.27	26.16 ± 13.23	23.4 ± 11.78	ND
ncPAHs	68.94 ± 20	69.13 ± 22.83	41.63 ± 18.15	39.92 ± 27.03	78.9 ± 44.44	63.67 ± 38.01	56.15 ± 26.02	46.66 ± 31.46
cPAHs	71.66 ± 30.89	73.59 ± 33.44	42.69 ± 10.43	44.9 ± 39.6	87.31 ± 48.78	61.22 ± 33.78	66.37 ± 33.99	60.36 ± 36.04
tPAHs	140.6 ± 49.53	142.72 ± 55.6	84.32 ± 22.02	84.82 ± 64.79	166.2 ± 90.16	124.89 ± 69.91	122.52 ± 55.67	107.02 ± 60.75
BaPeq	37.28 ± 13.19	35.7 ± 13.54	20.14 ± 6.27	22.1 ± 17.51	47.56 ± 23.55	33.86 ± 16.59	34.45 ± 16.41	27.55 ± 17.85

<u>The underlined PAHs</u> are recognized as carcinogenic.

Table S2. Beta-coefficients and 95 % confidence interval of Polycyclic aromatic hydrocarbons (PAHs) among non-cancer lung tissue (NC-NT), normal tissue from lung cancer patients (LC-NT), pre-cancerous tissues from lung cancer patients (LC-PC), and lung cancer tissue (LC-AC), adjusted by age, sex, smoking status, mask use, and occupation.

PAHs	LC-NT versus NC-NT	LC-PC versus NC-NT	LC-AC versus NC-NT	lung cancer versus non-lung cancer*
ACY	0.003 (-0.006, 0.011) P = 0.539	0.002 (-0.005, 0.009) P = 0.527	-0.005 (-0.010, 0.001) P = 0.081	-0.001 (-0.005, 0.004) P = 0.816
ACE	0.001 (-0.002, 0.003) P = 0.689	0.002 (-0.001, 0.004) P = 0.128	0.001 (-0.002, 0.003) P = 0.901	0.001 (-0.001, 0.002) P = 0.463
FLU	0.003 (-0.003, 0.008) P = 0.310	0.001 (-0.003, 0.006) P = 0.529	0.001 (-0.004, 0.005) P = 0.998	0.001 (-0.002, 0.004) P = 0.566
PHE	0.001 (-0.001, 0.003) P = 0.311	0.001 (-0.001, 0.003) P = 0.405	-0.001 (-0.003, 0.002) P = 0.556	0.001 (-0.001, 0.001) P = 0.968
ANT	0.002 (-0.001, 0.004) P = 0.137	0.001 (-0.002, 0.002) P = 0.992	0.001 (-0.002, 0.002) P = 0.952	0.002 (-0.001, 0.002) P = 0.701
FLA	0.001 (-0.001, 0.002) P = 0.369	0.001 (-0.002, 0.003) P = 0.638	-0.001 (-0.002, 0.002) P = 0.753	0.001 (-0.001, 0.001) P = 0.886
PYR	0.004 (-0.005, 0.013) P = 0.397	0.006 (-0.002, 0.013) P = 0.141	0.005 (-0.004, 0.130) P = 0.253	0.004 (-0.001, 0.009) P = 0.128
BaA	0.001 (-0.002, 0.004) P = 0.525	0.001 (-0.002, 0.004) P = 0.476	0.003 (-0.001, 0.006) P = 0.134	0.001 (-0.004, 0.003) P = 0.142
CHR	0.001 (-0.002, 0.004)	0.002 (-0.001, 0.005)	0.002 (-0.002, 0.005)	0.002 (-0.001, 0.003)

	P = 0.453	P = 0.205	P = 0.314	P = 0.077
BbF	0.011 (-0.001, 0.023)	0.008 (0.001, 0.015)	0.005 (-0.002, 0.012)	0.007 (-0.002, 0.013)
	P = 0.073	P = 0.042	P = 0.161	P = 0.013
BkF	0.006 (-0.002, 0.014)	0.004 (-0.003, 0.012)	0.004 (-0.002, 0.010)	0.004 (-0.001, 0.009)
	P = 0.133	P = 0.241	P = 0.186	P = 0.136
BaP	0.003 (-0.006, 0.011)	-0.001 (-0.008, 0.007)	-0.003 (-0.010, 0.004)	0.001 (-0.004, 0.004)
	P = 0.519	P = 0.914	P = 0.431	P = 0.893
IND	-0.006 (-0.017, 0.004)	-0.009 (-0.019, 0.002)	-0.011 (-0.022, 0.001)	-0.011 (-0.017, -0.005)
	P = 0.234	P = 0.092	P = 0.076	P = 0.001
DbA	0.015 (0.002, 0.028)	0.013 (0.002, 0.024)	0.012 (-0.001, 0.025)	0.013 (0.005, 0.020)
	P = 0.023	P = 0.021	P = 0.062	P = 0.001
BPER	0.019 (0.007, 0.030)	0.014 (0.004, 0.024)	0.014 (0.004, 0.025)	0.014 (0.009, 0.020)
	P = 0.003	P = 0.006	P = 0.008	P = <0.001
Total PAHS	0.062 (-0.002, 0.125)	0.045 (-0.009, 0.099)	0.026 (-0.030, 0.081)	0.035 (0.003, 0.068)
	P = 0.057	P = 0.097	P = 0.346	P = 0.034
Carcinogenic PAHS	0.029 (-0.006, 0.066)	0.019 (-0.011, 0.050)	0.012 (-0.022, 0.046)	0.016 (-0.004, 0.036)
	P = 0.100	P = 0.199	P = 0.462	P = 0.136
Non-carcinogenic PAHS	0.032 (0.001, 0.062)<br>P = 0.044	0.026 (-0.001, 0.052)<br>P = 0.056	0.013 (-0.012, 0.039)<br>P = 0.284	0.020 (0.005, 0.034)<br>P = 0.008

ACY; Acenaphthylene, ACE; Acenaphthene, FLU; Fluorene, PHE; Phenanthrene, ANT; Anthracene, FLA; Fluoranthene, PYR; Pyrene, BaA; Benz[a]anthracene, CHR; Chrysene, BbF; Benzo[b]fluoranthene, BkF;Benzo[k]fluoranthene, BaP;Benzo[a]pyrene, IND; Indeno[1,2,3-cd]pyrene, DbA; Dibenz[a,h]anthracene, BPER; Benzo[g,h.i] perylene

*analyzed by a Generalized Estimating Equations (GEE) model with a Gaussian family, identity link function, and exchangeable correlation structure, adjusted for age, sex, smoking status, mask use, and occupation.

Table S3. Association between Pack-year and total PAHs accumulation in each tissue in smoker patients analyzed by Generalized Linear Model (GLM) with a Poisson family and log link function

Study group	IRR	95% CI	p-value
Non-lung cancer tissue	0.99	0.91-1.10	0.961
Para-cancerous tissue	1.01	0.91-1.11	0.915
Lung cancer tissue	1.01	0.92-1.11	0.799

IRR; Incidence Rate Ratios

Table S4. Comparison of accumulated pulmonary PAHs (mean ± SD) between this study and previous literature.

Study	Description / Group	Sample Characteristics	PAHs concentrations (ng/g of wet tissue)															
			NaP	ACY	ACE	FLU	PHE	ANT	FLA	PYR	BaA	CHR	BbF	BkF	BaP	IND	DBA	BPER
Goldman R et al [27]	Lung tissue with cancer-free Caucasian and African-American autopsy donors	Cancer-free autopsy donors							0.151 ± 0.250	0.150 ± 0.293	0.055 ± 0.102	0.055 ± 0.105		0.039 ± 0.087	0.086 ± 0.216	0.088 ± 0.242	0.053 ± 0.149	0.110 ± 0.293
Cioroiu BI et al [26]	Lung tissue of Romanian patients with pulmonary cancer	Urban residents (n=16)	6.48 ± 9.60		8.95 ± 13.32	21.72 ± 45.92	1.17 ± 1.51	4.83 ± 7.57	5.31 ± 5.40	4.68 ± 451	3.57 ± 4.64	8.79 ± 10.44	2.01 ± 2.27	0.64 ± 0.75	0.63 ± 0.73	0.76 ± 1.28	1.16 ± 1.19	0.96 ± 0.84
		Rural residents (n=15)	2.53 ± 2.62		1.46 ± 2.34	10.67 ± 22.27	1.58 ± 1.94	1.89 ± 3.79	1.83 ± 2.50	3.02 ± 2.64	1.22 ± 1.45	3.65 ± 5.12	0.58 ± 0.62	0.25 ± 0.14	0.27 ± 0.20	0.19 ± 0.12	0.80 ± 1.09	0.48 ± 0.40
Pastor-Belda M et al [22]	Human lung tissue samples from autopsy			0.20 ± 0.53		0.66 ± 0.28	0.78 ± 0.51	0.21 ± 0.17		0.02 ± 0.03	0.06 ± 0.17*		0.61 ± 0.53	0.08 ± 0.07*		0.09 ± 0.22	0.12 ± 0.23	ND
Zhang X et al [19]	Lung tissues from Beijing-Tianjin-Hebei region, China	In the tissue	5.7	3.2	8.3	6.2	7.2	15.3	4.6	8.2	5.1	8	18.5	8.6	7.6	16.2	10.6	7.4
		In the pulmonary PM (μg/g)	2.9	1.4	8.6	3.5	6.9	11.2	1.2	5.6	1.5	2.7	9.8	3.8	2	9.6	7.4	0.6
This study	Lung tissue of Thai patients	Non-smoking patients																
		LC-NT (n = 5)		14.3 ± 4.38	ND	8.11 ± 2.87	4.91 ± 1.38	5.02 ± 1.2	5.12 ± 0.71	13.01 ± 8.9	5.45 ± 1.18	6.84 ± 0.86	20.01 ± 4.33	12.73 ± 1.1	13.42 ± 9.64	10.79 ± 0.99	23.34 ± 3.26	30.22 ± 8.09
		LC-PC (n = 5)		13.77 ± 2.38	8.25 ± 1.33	7.14 ± 2.36	5.24 ± 1.66	4.34 ± 0.49	5.08 ± 0.66	15.61 ± 12.01	5.3 ± 0.04	7.01 ± 0.78	16.9 ± 11.19	13.72 ± 5.24	14.67 ± 4.94	11.65 ± 6.67	23.6 ± 6.52	22.16 ± 5.09
		LC-AC (n=5)		10.08 ± 3.61	7.79	5.68 ± 2.37	4.07 ± 0.15	4.19 ± 1.08	3.65 ± 0.74	9.91 ± 4.15	4.36 ± 1.23	5.37 ± 1.21	7.9 ± 4.1	9.68 ± 2.83	7.25 ± 4.28	3.4 ± 0.9	15.01 ± 3.99	13.72 ± 3.19
		NC-NT (n=8)		12.57 ± 9.46	4.32 ± 0.52	7.6 ± 7.7	3.94 ± 2.09	4.69 ± 2.17	4.1 ± 1.76	12.37 ± 10.21	5.49 ± 3.32	3.94	12.42 ± 6.57	10 ± 8.2	14.8 ± 3.1	11.97 ± 9.63	19.12 ± 13.78	9.09 ± 1.53
		Smoking patients																
		LC-NT (n = 10)		21.69 ± 10.71	11.22	11.44 ± 5.91	6.75 ± 2.06	6.56 ± 2.10	5.88 ± 1.89	14.33 ± 13.73	12.33	9.86 ± 0.14	22.7 ± 18.69	16.8 ± 7.05	16.8 ± 7.19	22.39 ± 13.02	30.93 ± 15.36	32.34 ± 11.27
		LC-PC (n=10)		13.41 ± 7.6	9.03 ± 6.57	8.52 ± 4.63	4.65 ± 2.09	4.84 ± 2.32	5.76 ± 3.28	14.51 ± 8.06	5.61 ± 2.74	6.76 ± 3.50	12.26 ± 6.27	12.4 ± 7.78	13.04 ± 5.64	11.5 ± 11.19	23 ± 11.19	26.16 ± 13.23
		LC-AC (n=10)		6.14 ± 1.41	7.19 ± 1.95	6.89 ± 3.55	4.49 ± 2.26	4.36 ± 1.75	4.51 ± 1.73	16.31 ± 12.75	6.75 ± 2.95	9.18 ± 4.00	12.77 ± 5.76	11.23 ± 4.95	13.04 ± 6.59	23.13 ± 17.07	24.36 ± 11.31	23.4 ± 11.78
		NC-NT (n = 7)		17.35 ± 6.25	10.12 ± 5.00	7.79 ± 3.74	6.17 ± 4.34	5.46 ± 2.17	5.53 ± 1.90	13.04 ± 8.51	10.77	7.00 ± 0.31	18.76 ± 3.07	11.38 ± 5.66	21.48 ± 17.85	20.95 ± 14.31	27.39 ± 19.18	ND

*The values represent the sum of concentrations of two individual PAHs: BaA + CHR and BkF + BaP
